# Combination of Proton Pump Inhibitors and Food Elimination Diet for Eosinophilic Esophagitis Refractory to Monotherapy

**DOI:** 10.1016/j.gastha.2022.04.002

**Published:** 2022-04-14

**Authors:** John Leung, Twan Sia, Megan Miller, Evan Cunningham, Claire Buxton, Amy Huang, Daniel Pak, Sarah Johnson, Apaar Dadlani, Taylor Epstein, Kendall Garrett, Rebecca Nitschelm, Riki Tanaka, Thomas White, Kristen Park

**Affiliations:** 1 Division of Gastroenterology, Tufts Medical Center, Boston, Massachusetts; 2 Boston Specialists, Boston, Massachusetts

**Keywords:** PPI, FED, Histology, Eosinophils

## Abstract

**Background and Aims:**

Eosinophilic esophagitis (EoE) is an antigen-mediated inflammatory esophageal disease that is commonly treated with high-dose proton-pump inhibitors (PPIs), topical corticosteroids, or food elimination diet (FED) monotherapy. Combination treatment has not been well studied in the management of EoE. We aimed to determine if PPI and FED combination therapy was able to induce histologic remission in patients with EoE refractory to monotherapy.

**Methods:**

We conducted a retrospective cohort study identifying patients with EoE that was refractory to PPI monotherapy and FED monotherapy but histologically responsive to PPI and FED combination therapy. We also identified symptom changes through chart review.

**Results:**

Out of 405 EoE patients, 12 patients were identified with EoE that was refractory to PPI monotherapy and FED monotherapy but histologically responsive to PPI and FED combination therapy. Out of 12 patients, 11 (91.67%) noted resolution of symptoms while on combination therapy. Comparative analysis of peak eosinophil counts showed that patients achieved a median of 4.5 eos/hpf (interquartile range [IQR], 2–6.5), which was significantly decreased compared to baseline (median, 45; IQR, 35.5–50; Wilcoxon signed-rank test, *P* < .001), PPI monotherapy (median, 41; IQR, 26–50; Wilcoxon signed-rank test, *P* < .001), and FED monotherapy (median, 45; IQR, 17–67.5; Wilcoxon signed-rank test, *P* < .001).

**Conclusion:**

Our work shows that patients with EoE refractory to PPI monotherapy and FED monotherapy can successfully achieve histologic remission and symptom benefit with PPI and FED combination therapy. Therefore, combination therapy should be considered a viable option for patients with EoE who fail treatment with first-line monotherapies.

## Introduction

Eosinophilic esophagitis (EoE) is a chronic, allergen-mediated disease that is characterized by esophageal inflammation with mucosal eosinophilia. Active EoE is diagnosed with histologic findings of ≥15 eosinophils per high-power field (eos/hpf). Adults most commonly present with dysphagia, food impaction, and heartburn, while children tend to present clinically with vomiting, regurgitation, heartburn, and abdominal pain.^1^ High-dose proton-pump inhibitor (PPI) monotherapy is currently considered one of the first-line therapies and has been shown to be effective in inducing remission of EoE.^2^, ^3^, ^4^ Other effective treatments for EoE include various food elimination diets (FEDs) and topical corticosteroids.^2^^,^^5^, ^6^, ^7^

However, in some cases, monotherapy may not be able to induce histologic remission of EoE. Therefore, combination therapy, which still remains understudied, must be considered for a next line of treatment.^8^ Most literature studying combination therapy for EoE, however, has focused on topical corticosteroids and FED.^9^^,^^10^ In contrast, combination therapy of FED and PPI has rarely been discussed.^8^ A preliminary randomized controlled trial of 64 pediatric patients showed that a combination therapy of milk, wheat, soy, and egg FED (4-food elimination diet [4FED]) and PPI decreased esophageal eosinophil counts more than PPI monotherapy. Furthermore, they also showed that 4FED and PPI combination treatment induced histologic remission of EoE in a greater proportion of patients than PPI monotherapy.^11^ It is unclear, however, if a greater proportion of patients experience histologic remission in response to 4FED and PPI combination therapy compared to PPI monotherapy because a subset responds to PPI therapy while another subset responds to FED or if some patients are only responsive to combination therapy and not monotherapy. Therefore, following their work, we questioned if combination therapy of FED and PPI could benefit adult and pediatric patients who failed treatment with monotherapy of PPI and monotherapy of FED.

## Methods

### Study Design, Patients, and Measures

We conducted a retrospective cohort study of patients with EoE that was refractory to PPI and FED monotherapy but responsive to PPI and FED combination therapy. The International Classification of Diseases, Tenth Revision, (ICD-10) code K20.0 (eosinophilic esophagitis) was used to identify patients seen at a single academic center from January 2013 until September 2021 for chart review. Out of 405 patients with the ICD-10 code for EoE, patients were included in our analysis if they met the following criteria: (1) Diagnosis of EoE was made as defined by ≥15 eos/hpf, (2) documented histologically active EoE while on PPI monotherapy as defined by ≥15 eos/hpf, (3) documented histologically active EoE while on FED monotherapy, (4) histologic remission and reported symptom benefit of EoE while on combination therapy of PPI and FED, and (5) treatment plans on PPI monotherapy and FED monotherapy corresponded with the PPI and FED combination therapy that the patient followed. For FED, patients avoided eating suspected food triggers for their EoE, which varied from patient to patient. PPI therapy also varied between patients in terms of type and dosage. Histologic remission of EoE was defined by a peak eosinophil count of <15 eos/hpf in proximal, middle, and distal esophageal biopsies after esophagogastroduodenoscopies following at least 6 weeks of therapy. Clinical symptoms and peak eosinophil counts were abstracted from the electronic medical record. This study was approved by the Tufts Medical Center Institutional Review Board (ID: STUDY00002243).

### Statistical Analysis

Descriptive statistics were used to analyze the cohort characteristics and histoclinical features of the included patients. A Wilcoxon signed-rank test was used to compare peak eosinophil levels.

## Results

**Figure 1:**
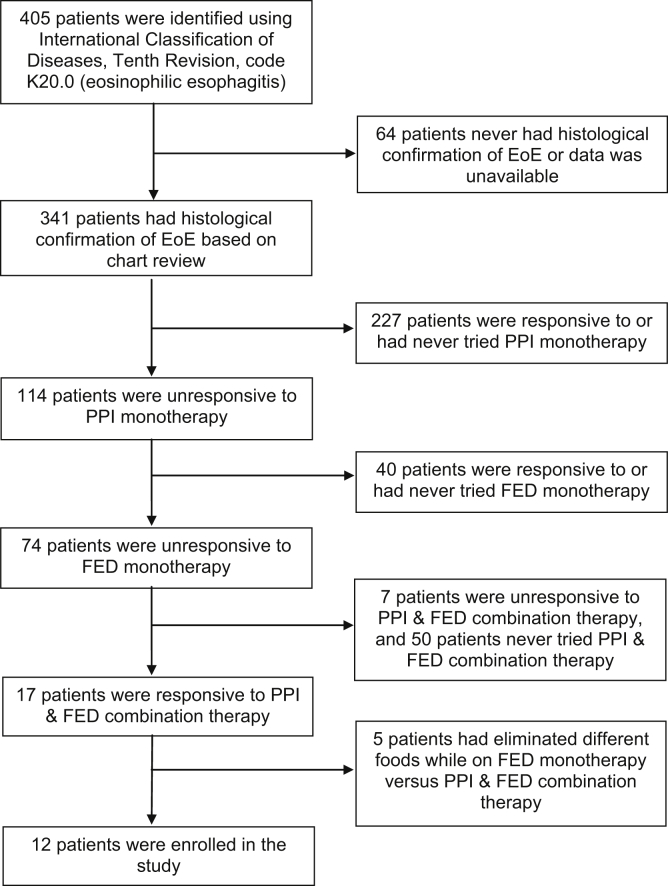
Flowchart of patients included in the study based on our inclusion criteria.

A total of 405 patients were identified with the ICD-10 code for EoE. Out of 405 patients, 341 patients had histologic confirmation of EoE based on a chart review by a physician. From this group, 227 patients had either never tried PPI monotherapy or were responsive to it. Therefore, they were excluded from this study. Out of 114 patients who had histologically confirmed EoE on PPI monotherapy, 40 patients had either never tried FED monotherapy or were responsive to it and were therefore excluded from our retrospective cohort. Out of the remaining 74 patients, 50 patients had never tried PPI and FED combination therapy, and 7 patients were unresponsive to it. Five patients were excluded from the study because they were on different elimination diets while on FED monotherapy vs PPI and FED combination therapy. Therefore, we identified 12 patients with EoE responsive to a combination therapy of PPI and FED but unresponsive to PPI monotherapy and FED monotherapy in our study (Figure 1).

Within our retrospective cohort of 12 patients, ages ranged from 7 to 53 years (median, 23; interquartile range [IQR], 16–36.5; Table 1), 8 patients were male (66.67%; Table 1), and 3 patients were pediatric (Table 1 and Table A1). Of 12 patients, 4 patients were diagnosed with histologically active EoE while on PPI monotherapy. Therefore, baseline characteristics are only available for 8 out of 12 patients. In this group, all were symptomatic with a majority of patients reporting dysphagia (87.5%; Table 2). Upon esophagogastroduodenoscopy, histologic findings showed a median peak eosinophil count of 45 eos/hpf (IQR, 35.5–50; Table 2).

**Table 1:** Demographics of Patients With EoE Refractory to PPI Monotherapy and FED Monotherapy but Responsive to PPI and FED Combination Therapy

Demographics	Patients responsive to PPI and FED combination therapy (n = 12)
Age, median (IQR)	23 (16–36.5)
Pediatric, n (%)	3 (25.0)
Male, n (%)	8 (66.67)
Atopic comorbidity, n (%)	
Any atopic condition	11 (91.67)
Atopic dermatitis	0 (0)
Allergic contact dermatitis	0 (0)
Asthma	5 (41.67)
Allergic rhinitis	9 (75.0)
Food allergy	7 (58.33)

**Table 2:** Histoclinical Characteristics of Patients Responsive to Combination Therapy of PPI and FED

Characteristics	Baseline (n = 8)^a^	PPI monotherapy (n = 12)	FED monotherapy (n = 12)	PPI and FED combination (n = 12)
Peak eos/hpf, median (IQR)	45 (35.5–50)	45.5 (35–61)	47.5 (17–70)	4 (0–5.5)
Symptoms, n (%)				
Dysphagia	7 (87.5)	9 (75.0)	7 (58.33)	0 (0)
Food impaction	1 (12.5)	0 (0)	0 (0)	0 (0)
Heartburn	5 (62.5)	3 (25.0)	5 (41.67)	1 (8.33)
Chest pain	0 (0)	0 (0)	0 (0)	0 (0)
Vomiting	2 (25.0)	0 (0)	1 (8.33)	0 (0)
Abdominal pain	0 (0)	0 (0)	0 (0)	0 (0)
Regurgitation	0 (0)	0 (0)	0 (0)	0 (0)
Asymptomatic	0 (0)	3 (25.0)	3 (25.0)	11 (91.67)

a Only 8 patients were diagnosed with EoE while they were not on a treatment plan. Therefore, descriptive statistics for baseline are calculated out of 8 total patients.

All 12 patients tried and failed treatment with PPI monotherapy. Different types of dosing schedules of PPIs were used, the most popular being omeprazole 40 mg twice daily (58.33%, Table 3 and Table A1). Following the 6 weeks of PPI monotherapy, 75% of patients were symptomatic with the majority of patients reporting dysphagia (75%; Table 2). In addition, patients reported continued symptoms of food impaction, heartburn, and abdominal pain among others (Table 2). Histologic findings on esophageal biopsies showed a median peak eosinophil count of 45.5 eos/hpf (IQR, 35–61; Table 2).

**Table 3:** Treatment Followed by Patients When Undergoing PPI Monotherapy, FED Monotherapy, and PPI and FED Combination Therapy

Treatment plans	Patients that tried each treatment plan (n = 12)
PPI monotherapy	
Omeprazole 40 mg twice daily	7 (58.33%)
Omperaozle 20 mg twice daily	3 (25.0%)
Esomeprazole 20 mg twice daily	1 (8.33%)
Omeprazole 10 mg twice daily	1 (8.33%)
FED monotherapy	
Milk, wheat, soy, egg, lentil, bean, chickpea FED	1 (8.33%)
Milk, wheat, soy, egg, coconut FED	1 (8.33%)
4FED	1 (8.33%)
Milk, wheat, corn FED	1 (8.33%)
2FED	4 (33.33%)
Wheat FED	1 (8.33%)
Milk FED	2 (16.67%)
Raw milk FED	1 (8.33%)
Combination therapy	
Omeprazole 40 mg twice daily & 2FED	2 (16.67%)
Omeprazole 40 mg twice daily & milk FED	3 (25.0%)
Omeprazole 40 mg twice daily & raw milk FED	1 (8.33%)
Omeprazole 40 mg twice daily & milk, wheat, soy, egg, lentil, bean, chickpea FED	1 (8.33%)
Omeprazole 20 mg twice daily & 2FED	2 (16.67%)
Omeprazole 20 mg twice daily & milk, wheat, soy, egg, coconut FED	1 (8.33%)
Esomeprazole 20 mg twice daily & milk, wheat, corn FED	1 (8.33%)
Omeprazole 10 mg twice daily & 4FED	1 (8.33%)

4FED Denotes milk, soy, egg, wheat FED, and 2FED denotes milk and wheat FED.

Additionally, all 12 patients tried and failed treatment with FED monotherapy. Patients adhered to different FEDs, with the most popular being a milk and wheat FED (2-food elimination diet; Table 3 and Table A1). Following 6 weeks of FED monotherapy, 75% of patients were symptomatic, with the majority of patients reporting dysphagia (58.33%; Table 2). Patients also reported continued symptoms of heartburn and vomiting, among other symptoms. Median peak eosinophils in esophageal biopsies were 47.5 eos/hpf (IQR, 17–70; Table 2).

Following failure to achieve histologic remission with PPI monotherapy and FED monotherapy as shown by peak eosinophil counts of ≥15 eos/hpf, all patients tried the PPI and FED combination therapy. All patients achieved histologic remission with a median peak eos/hpf of 4 (IQR, 0–5.5; Table 2). Patients adhered to different FEDs and dosing schedules of PPIs, with the most popular being omeprazole 40 mg twice daily with milk FED (25%; Table 3 and Table A1). Of all patients, 91.67% reported resolution of EoE symptoms although 1 patient reported continued dysphagia, and 2 others reported continued acid reflux (Table 2).

**Figure 2:**
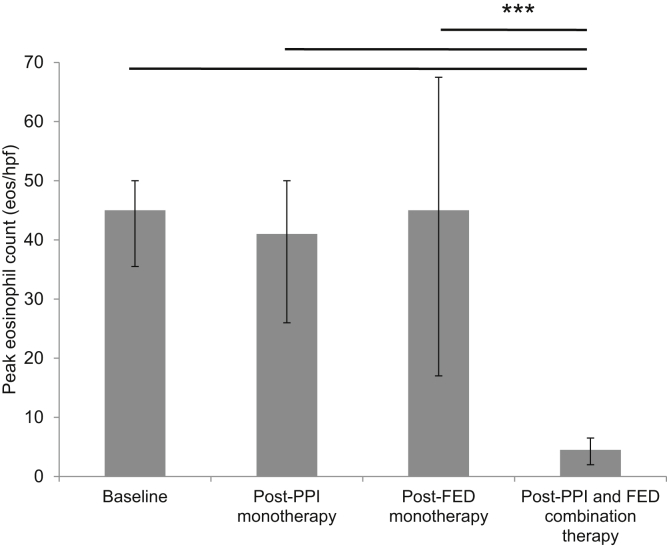
Peak eosinophils per high-power field in baseline (median, 45; IQR, 35.5–50) vs after PPI monotherapy (median, 41; IQR, 26–50), after FED monotherapy (median, 45; IQR, 17–67.5), and after PPI and FED combination therapy (median, 4.5; IQR, 2–6.5). Four patients were excluded due to being on PPIs at the time of EoE diagnosis. Error bars represent the interquartile range. Paired comparisons were made using the Wilcoxon signed-rank Test. ∗∗∗*P* < .001.

A comparative analysis was performed among the 8 patients who had baseline eosinophil counts available. Patients on the PPI and FED combination therapy had a median peak eosinophil count of 4.5 eos/hpf (IQR, 2–6.5), which was significantly less than that at baseline (median, 45; IQR, 35.5–50; Wilcoxon signed-rank test, *P* < .001), of those on PPI monotherapy (median, 41; IQR, 26–50; Wilcoxon signed-rank test, *P* < .001), and of those on FED monotherapy (median, 45; IQR, 17–67.5; Wilcoxon signed-rank test, *P* < .001; Figure 2).

## Discussion

Current guidelines on the management of EoE recommend PPI, FED, or swallowed topical corticosteroid monotherapy as a first-line option. If a particular first-line therapy fails to induce histologic remission, then the next choices include trialing a different alternative first-line monotherapy, elemental diet, or clinical trial drugs.^8^^,^^12^ Combination therapy has not been well studied in the context of EoE.^8^ In fact, combining FED and pharmacological therapy was considered to be improper as recently as 2017 due to the potential for additive side effects, negative impacts of quality of life, and confounding which treatment induces histologic remission.^13^

In our work, we identified 12 patients with EoE that was refractory to PPI monotherapy and FED monotherapy but was responsive to PPI and FED combination therapy. We highlight that combination therapy should be considered in the treatment algorithm for EoE, especially for patients that fail to achieve histologic remission with monotherapy.

Our study is limited by its retrospective nature, which prevented us from using certain analyzable endpoints such as validated symptoms outcome measures or endoscopic reference scores that are commonly used in EoE studies.^14^^,^^15^ In addition, our study contained a small number of patients. We were not able to identify the efficacy of the PPI and FED combination therapy in patients that had failed PPI monotherapy and FED monotherapy. Furthermore, a weakness of our work is that FED and PPI doses differed from patient to patient. Although we demonstrate that the combination therapy is effective with various permutations of FED and PPI, it is difficult to speculate about efficacy when much of the cohort is on different treatment plans. Therefore, further prospective work should consider standardized treatments.

In conclusion, through retrospective analysis, we provide preliminary evidence that patients’ EoE may be responsive to PPI and FED combination therapy, even if it is refractory to PPI monotherapy and FED monotherapy. To our knowledge, this study is the first to show the efficacy of the PPI and FED combination therapy in EoE refractory to PPI monotherapy and FED monotherapy. We provide evidence that combination therapy may be a helpful tool in treating EoE patients who have failed treatment with monotherapy. However, it is still imperative to consider the increased patient burden associated with combination therapy, which includes more demanding treatment plans, further endoscopic evaluations for histologic remission, and the potential for additive side effects. Therefore, further research investigating the risks and benefits associated with combination therapy for EoE may provide more information on whether a patient should consider it.

## Supplementary Materials

Table A1
