# Supplementary material for: Combination of Proton Pump Inhibitors and Food Elimination Diet for Eosinophilic Esophagitis Refractory to Monotherapy
Source: Gastro Hep Adv. 2022 Apr 14;1(4):596–600. doi: 10.1016/j.gastha.2022.04.002 (PMC11307866; doi:10.1016/j.gastha.2022.04.002)
Supplement: Table A1 [file mmc1.docx]

**Supplemental Table 1. Total patient characteristics.**

|  |  | **Baseline** | | | **Status post PPI monotherapy** | | | **Status post FED monotherapy** | | | **Status post-PPI and FED combination therapy** | | |
| --- | --- | --- | --- | --- | --- | --- | --- | --- | --- | --- | --- | --- | --- |
| **ID** | **Age/**  **Sex** | **Atopic conditions** | **Peak eos/hpf** | **Symptoms** | **Treatment**  **plan** | **Peak eos/hpf** | **Symptoms** | **Treatment plan** | **Peak eos/hpf** | **Symptoms** | **Treatment plan** | **Peak eos/hpf** | **Symptoms** |
| 1 | 39, Female | Allergic rhinitis | 33 | Dysphagia | Omeprazole 40 mg twice daily | 22 | Dysphagia | Raw milk FED | 15 | Dysphagia | Omeprazole 40 mg twice daily & Raw milk FED | 4 | Asymptomatic |
| 2 | 36, Male | Allergic rhinitis, asthma | 50 | Dysphagia, heartburn | Omeprazole 40 mg twice daily | 30 | Dysphagia | 2FED | 18 | Dysphagia, heartburn | Omeprazole 40 mg twice daily & Milk FED | 0 | Asymptomatic |
| 3 | 21, Male | Allergic rhinitis, food allergy | 30 | Dysphagia, heartburn | Esomeprazole 20 mg twice daily | 15 | Dysphagia, heartburn | Milk, wheat, corn FED | 40 | Dysphagia, heartburn | Esomeprazole 20 mg twice daily & milk, wheat, corn FED | 7 | Heartburn |
| 4 | 17, Male | Allergic rhinitis, asthma, food allergy | 40 | Dysphagia, vomiting | Omeprazole 40 mg twice daily | 45 | Dysphagia | 2FED | 50 | Dysphagia | Omeprazole 40 mg twice daily & 2FED | 5 | Asymptomatic |
| 5 | 37, Female | Allergic rhinitis | 50 | Dysphagia, heartburn | Omeprazole 40 mg twice daily | 42 | Dysphagia, heartburn | Milk, wheat, soy, egg, lentils, beans, chickpea FED | 80 | Dysphagia, heartburn | Omeprazole 40 mg twice daily & milk, wheat, soy, egg, lentils, beans, chickpea FED | 19 | Asymptomatic |
| 6 | 53, Male | Food allergy | N/A | N/A | Omeprazole 40 mg twice daily | 70 | Dysphagia, heartburn | Milk FED | 75 | Dysphagia, heartburn | Omeprazole 40 mg twice daily & milk FED | 4 | Asymptomatic |
| 7^†^ | 15, Male | No atopic conditions | 100 | Dysphagia, food impaction | Omeprazole 20 mg twice daily | 65 | Asymptomatic | Wheat FED | 75 | Asymptomatic | Omeprazole 20 mg twice daily & 2FED | 6 | Asymptomatic |
| 8 | 7, Male | Food allergy | 50 | Heartburn, vomiting | Omeprazole 10 mg twice daily | 55 | Dysphagia | 4FED | 60 | Asymptomatic | Omeprazole 10 mg twice daily & 4FED | 0 | Asymptomatic |
| 9 | 10, Female | Allergic rhinitis, asthma, food allergy | N/A | N/A | Omeprazole 20 mg twice daily | 57 | Dysphagia | Milk, soy, egg, wheat, coconut FED | 16 | Vomiting | Omeprazole 20 mg twice daily & milk, soy, egg, wheat, coconut FED | 0 | Asymptomatic |
| 10 | 35, Male | Allergic rhinitis | N/A | N/A | Omeprazole 40 mg twice daily | 75 | Asymptomatic | 2FED | 45 | Heartburn | Omeprazole 40 mg twice daily & 2FED | 0 | Asymptomatic |
| 11 | 25, Male | Allergic rhinitis, asthma, food allergy | N/A | N/A | Omeprazole 40 mg twice daily | 46 | Asymptomatic | Milk FED | 65 | Asymptomatic | Omeprazole 40 mg twice daily & milk FED | 2 | Asymptomatic |
| 12 | 21, Female | Allergic rhinitis, asthma, food allergy | 38 | Dysphagia, heartburn | Omeprazole 20 mg, twice daily | 40 | Dysphagia | 2FED | 16 | Dysphagia | Omeprazole 20 mg twice daily & 2FED | 4 | Asymptomatic |

Eosinophils per high-power field, eos/hpf; PPI, proton-pump inhibitor; FED, food elimination diet; 4FED, four-food elimination diet (milk, soy, egg, wheat FED); 2FED, two-food elimination diet (milk and wheat FED).

^†^Patient also had repeat EGD after 6 weeks of milk FED with a peak eosinophil count of 75 eos/hpf. Therefore, milk is also a trigger for this patient.
